# The Influence of Catechol-O-Methyltransferase Val158Met Polymorphism in Cognitive Performance and Executive Functioning in Women with Migraine

**DOI:** 10.3390/jcm15124551

**Published:** 2026-06-11

**Authors:** Margarita Cigarán-Méndez, Ana I. de-la-Llave-Rincón, Juan C. Pacho-Hernández, Angela Tejera-Alonso, Cristina Gómez-Calero, César Fernández-de-las-Peñas, Silvia Ambite-Quesada

**Affiliations:** 1 Department of Psychology, Universidad Rey Juan Carlos, 28922 Alcorcón, Spain; margarita.cigaran@urjc.es (M.C.-M.);; 2 Department of Physical Therapy, Occupational Therapy, Physical Medicine and Rehabilitation, Universidad Rey Juan Carlos, 28922 Alcorcón, Spain; anaisabel.delallave@urjc.es (A.I.d.-l.-L.-R.); cristina.gomez@urjc.es (C.G.-C.); silvia.ambite.quesada@urjc.es (S.A.-Q.); 3 Department of Research and Psychology in Education, Universidad Complutense de Madrid, 28040 Madrid, Spain; jpacho@ucm.es

**Keywords:** migraine, cognitive, executive function, Val158Met, Met allele, genetics

## Abstract

**Background/Objectives:** No study has investigated the effect of the catechol-O-methyltransferase (COMT) Val158Met rs4680 polymorphism in cognitive and executive performance in migraine. The current study investigated the potential influence of the Val158Met rs4680 polymorphism in cognitive performance/executive function in women with migraine. **Methods:** One hundred and forty women with migraine (70 chronic and 70 episodic) and 70 healthy controls completed the following neurocognitive tests (D2 Attention test and Rey–Osterrieth Complex Figure) and executive functions (subtest “Digits D/R/I” of the Wechsler Adult Intelligence Scale WAIS-IV battery for, the 5-Digit test, the Symbol Search for and the Zoo Test) for evaluating selective attention, visual perception, working memory, mental inhibition, processing speed and planning/decision making, respectively. Thus, three genotypes (Val/Val, Val/Met, and Met/Met) of the Val158Met polymorphism were identified by polymerase chain reaction. The effect of group and Val158Met genotype in neurocognitive tests and executive functions was evaluated with multivariate analysis of covariance (MANCOVA). **Results:** The MANCOVA revealed a significant Val158Met polymorphism* group interaction on neurocognitive performance (Wilk’s λ = 0.393, F [76,688] = 2.425, *p* < 0.001, n2p = 0.208, 1 − β = 0.999), not influenced by age (Wilk’s λ = 0.920, F [19,174] = 0.743, *p* = 0.734, n2p = 0.035, 1 − β = 0.120), educational level (Wilk’s λ = 0.875, F [19,174] = 1.024, *p* = 0.440, n2p = 0.047, 1 − β = 0.190) and prophylactic medication (Wilk’s λ = 0.855, F [19,174]= 1.000, *p* = 0.467, n2p= 0.145, 1 − β = 0.686). Post hoc analyses revealed that women with chronic migraine with the Met/Met genotype exhibited domain-specific better performance (i.e., higher selective attention, visuospatial memory) and executive functioning (i.e., working memory, planning/decision making) than those women with chronic migraine carrying Val/Val or Val/Met genotypes. **Conclusions:** We found an association of the Met/Met genotype with neurocognitive performance/executive functioning, particularly in women with chronic migraine since women with chronic migraine carrying the Met/Met genotype showed domain-specific better cognitive performance/executive functioning than those with the Val allele. Future studies including large sample sizes from different geographic locations are needed to better generalizability and validity of the current results.

## 1. Introduction

Migraine is a primary headache disorder showing worldwide pooled prevalence up to 34.6% [1,2]. In fact, migraine is the leading cause of work-disability in women younger than 50 years [3], and it is associated with social, work, economic and health impact to the society [4].

Since migraine is a multifactorial condition, multifactorial hypotheses are suggested to explain its pathogenesis [5,6]. One theory proposes the development of a cortical spreading depression (CSD) during migraine attacks which has been shown to be able of elevating the expression of proinflammatory biomarkers (e.g., cytokines, chemokines) and sensitize the trigemino-vascular system [7]. Thus, CSD has been used to explain the presence of cognitive alterations in this population [8], particularly during migraine attacks [9].

Cognitive alterations in people with migraine group heterogeneous symptoms range from memory deficit, concentration problems to lower mental processing speed [10,11]. In fact, cognitive deficits can be different according to the migraine phase (e.g., prodromic, migraine attack or resolution), although this assumption should be considered with caution [12]. In fact, data from published meta-analyses reveal heterogeneous results about the presence or absence of deficits in cognitive performance and executive functioning in people with migraine. Kaiser Pinotti et al. observed worse performance in domains such as attention, working memory, mental flexibility domains, and similar performance in others such as verbal fluency, inhibitory control, and response maintenance in individuals with migraine [11]. Pizer et al. reported worse performance in simple/complex attention, learning/memory, processing speed, visuospatial/construction, and language, but not in orientation, motor, and intelligence performance in patients with migraine [10]. Braganza et al. reported worse performance in complex attention, immediate/delayed memory, and spatial cognition in individuals with migraine even in the interictal phase [13]. Most reviews reported between-study heterogeneity and identified that most studies pooled data from individuals with episodic or chronic migraine, pooled data mixing men and women and did not control for covariables that can affect neurocognition [10,11,12,13].

A relevant aspect in migraine pathogenesis is the influence of genetics (alteration of several genes and single nucleotide polymorphisms) in altered nociceptive processing. More than 100 genes could be involved in chronic pain [14]. Among these genes, the catechol-O-methyltransferase (COMT) rs4680 is probably the single-nucleotide polymorphism (SNP) most investigated in chronic pain conditions [15] including migraine [16]. Nevertheless, evidence about an association of the Val158Met polymorphism and the risk of suffering from migraine is heterogeneous [17,18]. The lack of an association between Val158Met genotype and higher risk of developing migraine does not exclude a potential influence of this polymorphism in the clinical phenotype. In fact, preliminary evidence suggests that individuals with migraine carrying the Met allele experience worse clinical features and altered pain processing than those carrying the Val allele [19,20].

The COMT is an enzyme involved in metabolic degradation of several neurotransmitters such as dopamine, norepinephrine or epinephrine [21]. Since COMT has an important effect in frontal cortex dopamine function, a potential role of this gene in cognition is supported by animal studies showing that COMT genotype can predict executive functions related to info manipulation rather than to its info storage [22]. These results would suggest a domain-specific effect of the COMT gene on cognitive function. This cognitive domain-specificity would explain heterogeneity in the current literature. Old studies have initially reported that Met allele was associated with better performance on executive functions than the Val allele in healthy people [23,24]; however, pooled data from a posterior meta-analysis found no overall association between Val158Met genotype and executive functions [25]. More recent studies have observed that the Val158Met genotype can be associated with some executive functions. For instance, Khanthiyong et al. identified that people carrying the Met allele exhibited better cognitive set shifting, but not other domains (assessed with the Wisconsin Card Sorting Test), than those subjects carrying the Val allele and that this association was influenced by age and educational levels [26]. Frois et al. reported that the response time on a task assessing work memory, inhibitory control, and cognitive flexibility of Val allele carriers was shorter than that of Met allele carriers [27]. Some studies have detected that the effect of COMT is age-dependent since Val/Val carriers have a detrimental effect on cognitive performance in older, but not young, people [28,29].

Most of the previous studies have included healthy pain-free populations. No study has investigated the effect of the COMT gene in cognitive performance/executive functions in migraine. Accordingly, this study investigated the influence of the Val158Met rs4680 polymorphism in cognitive performance and executive functions in migraine individuals. Since the effect of the COMT gene on cognitive function is age- and sex-dependent [28,29,30], and because migraine is more prevalent [31] and provokes double the health loss [32] in women than in men, this research included middle-aged women with migraine [33]. Thus, since the Met allele of the rs4680 COMT polymorphism produces low gene activity which reduces degradation rate and, hence, increases cortical dopamine levels [34], we hypothesized that women (without or with migraine) carrying the Met allele will exhibit better overall cognitive performance and executive functioning than women carrying the Val allele.

## 2. Methods

### 2.1. Participants

This investigation was designed as a case–control study and followed the recommendations of the Strengthening the Reporting of Observational Studies in Epidemiology (STROBE) statement [35]. Women with migraine (episodic or chronic) were consecutively recruited from the Headache Unit of the Neurology Department at Hospital Universitario Fundación Alcorcón (HUFA), an urban hospital in Madrid, Spain. Diagnosis of migraine was established by an experienced neurologist according to the current criteria of the International Headache Society [36]. Data obtained from medical records included pain distribution and characteristics, years since migraine onset, headache frequency and intensity, family history, and medication use. Each participant also kept a headache diary for 4 weeks, documenting: monthly number of migraine days, duration of attacks in hours, and intensity rated on a Numerical Pain Rating Scale (NPRS, 0–10) [37].

Women were excluded if they: 1, had any additional primary or secondary headache disorder [36]; 2, reported previous cervical trauma (e.g., whiplash) or cervical disc herniation; 3, suffered from other medical conditions altering nociceptive processing (e.g., fibromyalgia); 4, presented with any psychiatric disorder diagnosed according to DSM-V (e.g., schizophrenia, neurocognitive disorders); 5, were taking medications with cognitive effects (antipsychotics, anticonvulsants, anticholinergics) [38]; 6, were pregnant; or 7, had received therapeutic interventions, including anesthetic blocks, in the last 3 months.

A control (non-migraine) group consisting of age-matched women without a history of migraine or recurrent headaches and who had not experienced a headache episode within the previous 12 months was recruited from local announcement. Eligibility was confirmed by neurological clinical examination.

The study has been approved by Local Ethics Committees of the institutions involved (HUFA 24_117; URJC_010220240912024). All participants were informed of the study and signed the informed consent before their enrollment. All procedures were performed following the ethical standards of the Declaration of Helsinki.

In women with episodic migraine, the evaluation was performed when they were headache-free and when at least one week had elapsed since the last attack. In those women with chronic migraine, evaluation was conducted pain-free (if possible) or when the headache intensity was <3/10 points. Thus, evaluation was tried to be at least one–two days since the last headache. All participants were asked to avoid taking analgesics or muscle relaxants 48 h prior to the examination.

### 2.2. Selection Attention

The Spanish version of the D2 Attention test (D2) [39] was used for measuring selective attention and mind concentration [40]. The D2 test includes 14 lines of 47 characters each (total 658 items). Within each 20 s row, participants must mark all “d” symbols with exactly two dashes (both above, below or one above/one below), while ignoring distractors (e.g., “p” or “d” with incorrect number of dashes). The test usually lasts between 8 and 10 min. The scores obtained include: D2_TR, total number of elements; D2_TA, the number of relevant elements marked; D2_O, number of relevant elements not marked; D2_C, number of irrelevant elements marked; D2_TOT, total test effectiveness, TR - (O+C); D2_CON, concentration index, TA-C; D2_TR+, line with a greater number of elements tried; D2_TR-, line with a lower number of elements tried; and D2_VAR, variation index or difference, TR+ (-) TR-.

### 2.3. Visuospatial Memory

The Rey–Osterrieth Complex Figure (ROCF) was used for measuring visual perception, visuo-constructional ability and spontaneous memory retention [41,42]. First, participants copy a geometric figure consisting of 18 black lines on a paper. Second, the participant must redraw the figure immediately afterwards (immediate recall) and after 20–30 min (delayed recall). No instructions to memorize the figure are provided. Data from this test include: copy, immediate recall and delayed recall points (one point for accuracy and location/unit and expressed as percentage: copy points/maximum points; ROCF_Copy); recall percentage (delayed recall points/immediate recall points, ROCF_Recall), and the time needed for doing the copy (ROCF_TimeCopy).

### 2.4. Executive Functions

“Working memory” was tested with the “Digits D/R/I” subtest of the Wechsler Adult Intelligence Scale (WAIS-IV) battery [43]. The following three conditions are coded: digit span forward (DSF, repetition of the digits in the same order); digit span backward (DSB, repetition of digits in reverse order); digits span sequencing (DSS, repetition of digits in ascending order).

“Mental inhibition” was evaluated with the “response inhibition index” of the 5-Digit test (FDT), STROOP-type task [44]. It includes four blocks of 50 items evaluating Reading, Counting, Election and Alternation. Reading/Counting assess basic automatic skills, while Election/Alternation tap higher-level inhibition and flexibility [44]. Two complementary scores, one for response inhibition and other for mental flexibility, combining errors and time to completion are obtained [44]. Specific scores are: Decoding_FDT (seconds to read all numeric-items); Retrieving_FDT (seconds to read all non-numeric items such as asterisks); Inhibiting_FDT (seconds to repeat identical numeric items); and Shifting_FDT (seconds to read the group of the same numeric item mixed with numeric items within a box).

“Processing speed” was assessed through the “Symbol Search” (SS) subtest of the WAIS-IV battery [45]. Participants are given a key area with nine digit–symbol pairs and a response area with numbers alongside empty spaces. They must insert the matching symbols as fast as possible within 120 s.

“Planning/decision making” was scored with the Zoo Map Test [46]. The first part of the test evaluates planning without a fixed structure, while the second part requires applying an external strategy. In each part, errors are subtracted from the sequence score. A total score between 0 and 16 is calculated by summing both parts [46].

### 2.5. DNA Collection and COMT Genotyping

To ensure a non-invasive, stress-free, and ethically suitable assessment, salivary sampling was prioritized over blood collection. Unstimulated saliva was obtained via the passive drooling technique using specific collection tubes in accordance with standardized protocols. Participants refrained from eating, drinking, or chewing gum for one hour prior to collection. Immediately following recovery, the samples were centrifuged at 3000 rpm for 15 min to isolate the cell sediment and subsequently stored at −20 °C until the primary analysis. Laboratory technicians remained blind to the participants’ clinical conditions.

Genomic DNA was extracted from the salivary cell pellets using the “Genomic DNA extraction and purification Kit” (Real Molecular Biology, REAL Laboratory, Durviz, Valencia, Spain) according to the manufacturer’s protocol. Genotyping of the single Val158Met rs4680 nucleotide polymorphism was performed using TaqMan^®^ Drug Metabolism Genotyping Assays on an ABI Prism 7000 Real-Time PCR Sequence Detection System (Applied Biosystems, New York, NY, USA). This analysis was conducted at the Genomics Unit of the Centro de Apoyo Tecnológico, Universidad Rey Juan Carlos, Madrid, Spain. Distinct fluorescent dyes were coupled with the respective alleles to guarantee the identification of the three potential genotypes: Val/Val (H/H), Val/Met (H/L), and Met/Met (L/L). These variants result from a G→A transition within the following sequence:

CCAGCGGATGGTGGATTTCGCTGGC [A/G] TGAAGGACAAGGTGTGCATGCCTGA

### 2.6. Statistical Analysis

All statistical evaluations were performed utilizing the SPSS software version 25.0 package. Boxplot examinations were executed to identify potential data outliers, while the underlying assumptions of normality and sphericity were systematically verified. Quantitative variables are expressed as means accompanied by their respective standard deviations, whereas categorical data are presented using frequencies alongside percentages.

A multivariate analysis of covariance (MANOVA/MANCOVA) to determine the effect of group (episodic migraine, chronic migraine, pain-free women) and Val158Met genotype (Val/Val, Val/Met, Met/Met) on neurocognitive tests and executive functions was conducted. Since age, education level and medication have a significant association with the Val518Met polymorphism, these variables were introduced as covariates into the MANCOVA. For post hoc analyses, *p* value < 0.015 (0.05/3) was the level considered as statistically significant (correction for multiple comparisons). Thus, effect sizes were calculated with partial eta squared (n2p), where a value of 0.01 represents a small effect, a value of 0.06 represents a medium effect, and values greater than 0.14 represent a large effect [47].

## 3. Results

### 3.1. Descriptive and Data Distribution of Val158Met Polymorphism in Migraine

Sociodemographic features, clinical variables, and the distribution of the Val158Met genotype can be seen in Table 1. The Hardy–Weinberg equilibrium was verified for distribution of Val158Met genotypes. No significant difference in distribution of Val158Met genotype (*p* = 0.097) was seen between women with episodic/chronic migraine and controls.

### 3.2. Val158Met Polymorphism and Neurocognitive Performance

The MANCOVA revealed a significant effect of Val158Met polymorphism (Wilk’s λ = 0.624, F [38,348] = 2.431, *p* < 0.001, n2p = 0.210, 1 − β = 0.999), group (Wilk’s λ = 0.448, F [38,348] = 4.522, *p* < 0.001, n2p = 0.331, 1 − β = 0.999), and a significant group*Val158Met polymorphism interaction on neurocognitive performance (Wilk’s λ = 0.393, F [76,688] = 2.425, *p* < 0.001, n2p = 0.208, 1 − β = 0.999), after controlling for age (Wilk’s λ = 0.920, F [19,174] = 0.743, *p* = 0.734, n2p = 0.035, 1 − β = 0.120), educational level (Wilk’s λ = 0.875, F [19,174] = 1.024, *p* = 0.440, n2p = 0.047, 1 − β = 0.190), and prophylactic medication (Wilk’s λ = 0.855, F [19,174] = 1.000, *p* = 0.467, n2p= 0.145, 1 − β = 0.686).

Post hoc analyses showed that women with chronic migraine with the Met/Met genotype exhibited better cognitive performance/executive functioning as expressed as: (1) higher scores in d2_TA and d2_CON than those women with chronic migraine with the Val/Val (d2_TA, between-groups difference: 34 points, 95%CI 5.9 to 62.1, *p* = 0.011; d2_CON, mean difference: 37.2 points, 95%CI 6.5 to 67.9, *p* = 0.011) and Val/Met (d2_TA, between-groups difference: 33.4 points, 95%CI 10.0 to 56.8, *p* = 0.002; d2_CON between-groups difference: 34.6 points, 95%CI 9.0 to 60.1, *p* = 0.004) genotype; (2) lower score in d2_O than women with the Val/Val (between-groups difference: −36.2 points, 95%CI −59.8 to −12.7, *p* = 0.001) and Val/Met (between-groups difference: −34.7 points, 95%CI −54.3 to −15.1, *p* < 0.001) genotypes; (3) higher score in d2_VAR than women with the Val/Val genotype (between-groups difference: 6.0 points, 95%CI 0.6 to 11.5, *p* = 0.014); (4) lower scores in DSF (between-groups difference: −5.2 points, 95%CI −2.7 to −0.3, *p* = 0.006) than those with the Val/Met genotype; (5) higher score in ROCF_Copy than women with the Val/Met genotype (between-groups difference: 3.4 points, 95%CI 0.6 to 6.3, *p* = 0.011) and in ROCF_Recall than women with the Val/Val genotype (between-groups difference: 5.9 points, 95%CI 1.4 to 10.4, *p* = 0.005); and (6) higher score in the Zoo Map test (between-groups difference: 2.9 points, 95%CI 0.9 to 4.9, *p* = 0.002) than women with the Val/Met genotype (Table 2). Univariate tests with the estimated marginal means and standard deviations of women with chronic migraine are shown in Table 2.

The results of the univariate tests, together with the estimated marginal means and standard deviations within women with episodic migraine are shown in Table 3. Pairwise comparisons revealed some significant trends, but without reaching the threshold due to the Bonferroni correction. In such a scenario, women with episodic migraine carrying the Met/Met genotype tend to exhibit better cognitive performance by showing higher scores in ROCF_Recall than patients those carrying the Val/Val genotype (between-groups difference: 4.3 points, 95%CI 0.1 to 8.6, *p* = 0.041), and higher scores in Symbol Search than those carrying the Val/Met genotype (between-groups difference: 5.8 points, 95%CI 0.3 to 11.2, *p* = 0.031, Table 3).

Post hoc analyses showed that women with the Val/Met genotype exhibited better cognitive performance expressed as higher scores in d2_TR than those with the Met/Met genotype (between-groups difference: 86.7 points, 95%CI 13.2 to 160.1, *p* = 0.015), and higher scores in d2_TA (between-groups difference: 32.1 points, 95%CI 8.2 to 56.0, *p* = 0.004), d2_CON (between-groups difference: 31.0 points, 95%CI 4.9 to 57.0, *p* = 0.014); d2_TOT (between-groups difference: 56.0 points, 95%CI 3.7 to 108.2, *p* = 0.031) and Zoo Map test (between-groups difference: 1.9 points, 95%CI 0.1 to 4.0, *p* = 0.049) than women carrying the Val/Val genotype (Table 4). Univariate tests with the estimated marginal means and standard deviations of healthy controls are summarized in Table 4.

## 4. Discussion

The current study observed an association of the Val158Met polymorphism, particularly the Met/Met genotype, with neurocognitive performance and executive functioning. This association was evident in women with chronic, but not episodic, migraine. Women with chronic migraine carrying the Met/Met genotype exhibited better cognitive performance/executive functioning in some domains than those women with the Val/Val or Val/Met genotype. Specifically, women with chronic migraine carrying the Met/Met genotype had higher scores in selective attention, visuospatial memory, working memory as well as planning/decision making than women with chronic migraine carrying the Val/Val or Val/Met genotype. No significant association between any Val158Met genotype and any doming of neurocognitive or executive function was found in episodic migraine.

The presence of neurocognitive and executive functioning impairment in migraine is not new; nevertheless, the literature supports that this migraine-associated “brain fog” is domain-specific. For instance, cognitive domains which have been found to be impaired in migraine include attention, working memory, processing speed, mental flexibility and visuospatial construction [10,11,12,13]. Other domains such as inhibitory control, response, and intelligence exhibit similar performance between individuals with/without migraine [10,11,12,13]. It should be noted that most reviews have reported between-study heterogeneity and identified that most papers did not differentiate between patients with episodic or chronic migraine; they included men and women and did not control for those variables affecting cognition [10,11,12,13]. A recent meta-analysis has also confirmed the specificity of cognitive performance impairment in this population since migraine is not associated with a global memory deficit, but rather with a working memory selective impairment [48]. These authors suggested that migraine could mainly affect cognitive performance in domains requiring higher demands on attentional control and mental effort and not as much as those domains assessing storage capacity under minimal interference [48].

Cognitive dysfunction in migraines is multidimensional, involving intrinsic mechanisms (e.g., brainstem neural dysfunction) and external modulators (e.g., stress and pain). Since COMT gene has an important effect in cortex dopamine function, its function can predict cognitive performance and executive functions related to info manipulation [22]. To the best of our knowledge, this is the first study that has preliminary identified a relationship between the Met/Met genotype and neurocognitive/executive functioning performance in migraine. We found that women with chronic migraine carrying the Met/Met genotype had higher performance in selective attention, visuospatial/working memory and planning/decision making than women with chronic migraine carrying the Val/Val or Val/Met genotype. No association of any Val158Met genotype with neurocognitive/executive functioning performance was identified in women with episodic migraine. Our results agree with those previously reported in people with schizophrenia where Met/Met carriers exhibited better performance in verbal/visual memory, information processing speed, and regulatory functions when compared with Val/Val carriers [49]. Accordingly, current and previous findings suggest that Val158Met polymorphism has a domain-specific effect of cognitive/executive functioning performance, since not all domains are affected.

The Met/Met genotype has been previously associated with better performance in domains of working memory requiring storage and manipulation of information, but similar performance in other domains assessing simple storage, maintenance of temporal order or updating of information in working memory in healthy individuals [23]. In fact, it seems that the Met allele is associated with increased dopamine levels in the prefrontal cortex, which could lead to better performance on working memory tasks [50]. Surprisingly, we did not find better cognitive performance in the Met/Met genotype in our sample of pain-free healthy women. Thus, we observed that pain-free healthy women carrying the Met/Met genotype exhibited similar or even worse specific-domain cognitive performance (e.g., attention, planning/decision making) than pain-free healthy women with the Val/Val or Val/Met genotype. Bruder et al. suggested that domains involving higher-order components of processing (e.g., mental manipulation) could be more closely related to the Val158Met polymorphism [23]. We should also recognize that the prevalence rate of the presence of the Met/Met genotype in our healthy pain-free group was just 14%. In fact, normative prevalence of the Met/Met genotype in healthy people varies significantly by ethnicity, ranging from 10% to 37% [51]. It is possible that the lower prevalence rate of the Met/Met genotype in our control group could have led to a Type II error.

Interestingly, we observed better cognitive/executive functioning performance in women with chronic, but not episodic, migraine carrying the Met/Met genotype. The lack of significant differences in the distribution of Val158Met genotypes between women with episodic/chronic migraine and healthy controls cannot explain these results. The effect of Met/Met genotype in the clinical phenotype of migraine has been found to be present within the chronic, but not the episodic, form of the disease. In a previous study, women with chronic, but not episodic, migraine carrying the Met/Met genotype exhibited higher anxiety/depressive levels and widespread pressure pain hyper-sensitivity than those carrying the Val/Val or Val/Met genotype [20]. It is suggested that some underlying mechanisms explaining migraine-associated cognitive impairment include abnormal synaptic plasticity and thalamocortical dysfunction, mechanisms which have shown correlation with genetic polymorphisms [52]. Thus, maladaptive neuroplasticity in brain areas responsible for nociception and cognition may be more vulnerable in those subjects carrying the Met/Met genotype and, in chronic migraine patients, a cumulative effect due to frequent attacks on pre-frontocerebellar networks [53] can explain a higher interaction between COMT gene and neurocognitive performance.

Although this is the first study to investigate the association between the Val158Met polymorphism and cognitive/executive performance in patients with migraine, several limitations must be acknowledged. First, the cross-sectional design precludes the establishment of definitive causal relationships. Second, while conducting multiple comparisons inherently increases the risk of a Type I error, we mitigated this by employing restricted multivariate analyses adjusted via Bonferroni correction. Third, our sample consisted exclusively of middle-aged women with/without migraine. Given the female predominance in migraine prevalence, alongside known age- and sex-dependent variations in cognitive profiles, these findings should not be extrapolated to male populations. Thus, we only included migraine without aura. Thus, we do not currently know the effects of this polymorphism on cognitive performance and executive functions would be different in women with migraine with aura. Fourth, this study was conducted in a single center, e.g., specialized tertiary hospital center, in Spain which could introduce geographic/ethnic bias. Fifth, although we did not observe differences in prophylactic medication intake between women with episodic/chronic migraine and no significant effect was identified, it should be considered that prophylactic medication like amitriptyline has anticholinergic effects, and even low-dose amitriptyline may affect attention and memory domains. Thus, patients actively prescribed psychoactive drugs or medications known to interfere with cognitive processing were strictly excluded. Sixth, our investigation was confined to a single nucleotide polymorphism. Future research should encompass a broader array of genetic variants and candidate genes to comprehensively elucidate their contribution to executive and cognitive functioning in migraine. Finally, the neuropsychological battery employed might lack the sensitivity required to detect subtle, migraine-specific cognitive deficits. Indeed, the previous literature documenting cognitive impairments in this population relies on highly heterogeneous testing protocols [10,11,12,13]. Therefore, establishing standardized neuropsychological instruments optimized for the specific cognitive domains affected in migraine patients remains a critical necessity [54]. Accordingly, future studies including large sample sizes from different geographic locations and considering all the limitations identified in this study are needed to extrapolate and validate current results.

## 5. Conclusions

This study found a preliminary domain-specific association of rs4680 Val158Met polymorphism, particularly the Met/Met genotype with neurocognitive performance and executive functioning in women with chronic migraine. Women with chronic, but not episodic, migraine carrying the Met/Met genotype exhibited domain-specific better performance (i.e., selective attention, visuospatial memory) and executive functioning (i.e., working memory, planning/decision making) than women with chronic migraine carrying the Val/Val or the Val/Met genotype. No association of COMT genotype with neurocognitive/executive functioning performance was observed in women with episodic migraine. Future studies including large sample sizes from different geographic locations are needed to confirm and validate current results.

## Figures and Tables

**Table 1:** Clinical features and Val158Met genotype distribution of the sample.

	Chronic Migraine (n = 70)	Episodic Migraine (n = 70)	Controls (n = 70)		
	Mean (SD)	Mean (SD)	Mean (SD)	F	*p*
Age (years)	46.1 (5.2)	48.1 (7.2)	49.1 (9.6)	2.905	0.057
Intensity (NPRS, 0–10)	8.6 (1.4)	7.6 (1.55)	---		
Frequency (days/month)	16.5 (6.4)	3.5 (2.7)	---		
Duration (hours/attack)	33.1 (20.3)	25.3 (23.9)	---		
	**n (%)**	**n (%)**	**n (%)**	**χ^2^**	***p***
Side of migraine Left Right Bilateral					
	12 (17%) 19 (27%) 39 (56%)	14 (20%) 17 (24%) 39 (56%)	--- --- ---	1.602	0.445
Educational level Primary Secondary Higher education	0 (0%) 31 (44.3%) 39 (55.7%)	4 (5.7%) 16 (22.9%) 50 (71.4%)	4 (5.7%) 14 (20%) 52 (74.30%)	14.577	0.006
Preventive Treatment Yes (Amitriptyline) No	52 (74.3%) 18 (25.7%)	48 (68.6%) 22 (31.4%)		0.560	0.454
Val158Met Genotype Val/Val Val/Met Met/Met	15 (21.43%) 31 (44.28%) 24 (34.29%)	21 (30.00%) 28 (40.00%) 21 (30.00%)	21 (31.25%) 38 (54.69%) 11 (14.06%)	7.856	0.097

n: number of subjects, SD: standard deviation.

**Table 2:** Effect of Val158Met genotype on neurocognitive performance and executive functions in women with chronic migraine (n = 70).

	Val/Val (n = 15)	Val/Met (n = 31)	Met/Met (n = 24)				
	Mean (SD)	Mean (SD)	Mean (SD)	F	*p*	n^2^p	β − 1
d2_TR	439.5 (21.1)	435.1 (14.9)	436.7 (16.6)	0.014	0.986	0.000	0.052
d2_TA *	139.3 (9.1)	139.9 (6.4)	173.4 (7.2)	7.173	0.001	0.070	0.930
d2_O *	44.3 (7.6)	42.8 (5.3)	8.0 (6.0)	11.264	<0.0001	0.105	0.992
d2_C	3.4 (1.7)	1.7 (1.2)	0.3 (1.3)	1.044	0.354	0.011	0.231
d2_TOT	391.7 (20.0)	390.5 (14.1)	428.3 (15.7)	1.849	0.160	0.019	0.382
d2_CON *	135.7 (10.0)	138.3 (7.0)	172.9 (7.8)	6.724	0.002	0.065	0.913
d2_VAR *	10.2 (1.7)	12.6 (1.2)	16.2 (1.4)	4.270	0.014	0.040	0.707
DSF *	8.2 (0.4)	8.6 (0.3)	7.0 (0.3)	5.254	0.006	0.052	0.829
DSB	7.0 (0.4)	7.2 (0.3)	8.2 (0.3)	3.355	0.037	0.034	0.628
DSS	7.4 (0.5)	8.1 (0.3)	7.3 (0.4)	1.448	0.238	0.015	0.307
ROCF_Copy *	29.8 (1.1)	29.6 (0.7)	33.1 (0.8)	4.908	0.008	0.049	0.801
ROCF_Recall *	9.2 (1.4)	13.1 (1.0)	15.2 (1.1)	5.066	0.007	0.050	0.814
ROCF_TimeCopy	2.0 (0.2)	2.6 (0.1)	2.1 (0.1)	2.732	0.068	0.028	0.535
Symbol Search	28.4 (2.0)	33.1 (1.4)	34.3 (1.5)	2.783	0.064	0.028	0.543
Decoding_FDT	19.0 (0.9)	19.9 (0.6)	21.8 (0.7)	2.928	0.056	0.030	0.566
Retrieving_FDT	22.7 (1.3)	25.9 (0.9)	23.1 (1.0)	2.707	0.069	0.027	0.531
Inhibiting_FDT	32.6 (2.0)	37.1 (1.4)	31.8 (1.6)	3.261	0.040	0.033	0.615
Shifting_FDT	41.8 (2.6)	45.1 (1.8)	38.6 (2.0)	2.763	0.066	0.028	0.540
Zoo Map test *	14.6 (0.7)	11.7 (0.5)	14.6 (0.6)	7.564	<0.0001	0.073	0.942

d2_TR = total number of items answered; d2_TA = number of items answered correctly; d2_O = errors of omission committed; d2_C = commission errors made; d2_TOT = number of elements processed minus the total number of errors committed; d2_CON = number of relevant elements marked minus the number of commissions; d2_VAR = variation index d2; DSF = Digit Span Forward; DSB = Digit Span Backward; DSS = Digit Span Sequencing; ROCF_Copy = direct scoring in the copy phase of the Rey–Osterrieth Complex Figure; ROCF_Recall = direct scoring in the delayed Recall phase of the Rey–Osterrieth Complex Figure; Symbol Search = direct scoring of correctly answered items; Decoding_FDT = time in seconds to read all numeric items; Retrieving_FDT = time in seconds to read all non-numeric items; Inhibiting_FDT = time in seconds to read numeric items; Shifting_FDT = time in seconds to read non-numeric items; Zoo Map Test = direct score in carrying out the planning test. * Statistically significant different by genotype (*p* < 0.015).

**Table 3:** Effect of Val158Met genotype on neurocognitive performance and executive functions in women with episodic migraine (n = 70).

	Val/Val (n = 21)	Val/Met (n = 28)	Met/Met (n = 21)				
	Mean (SD)	Mean (SD)	Mean (SD)	F	*p*	n^2^p	β − 1
d2_TR	410.4 (17.9)	419.5 (15.7)	407.0 (17.7)	0.153	0.858	0.002	0.073
d2_TA	144.7 (7.7)	144.7 (6.7)	138.1 (7.6)	0.256	0.774	0.003	0.090
d2_O	27.3 (6.4)	31.3 (5.6)	34.2 (6.4)	0.285	0.753	0.003	0.095
d2_C	4.4 (1.4)	5.3 (1.2)	5.7 (1.4)	0.219	0.804	0.002	0.084
d2_TOT	378.6 (16.9)	383.0 (14.8)	367.0 (16.7)	0.263	0.769	0.003	0.091
d2_CON	138.9 (8.4)	138.1 (7.4)	131.3 (8.3)	0.254	0.776	0.003	0.090
d2_VAR	17.2 (1.5)	15.5 (1.3)	14.6 (1.4)	0.774	0.462	0.008	0.181
DSF	8.5 (0.3)	7.9 (0.3)	7.7 (0.3)	1.099	0.335	0.011	0.241
DSB	6.7 (0.3)	7.0 (0.3)	7.1 (0.3)	0.244	0.783	0.003	0.088
DSS	9.0 (0.4)	8.6 (0.3)	8.0 (0.4)	1.323	0.269	0.014	0.284
ROCF_Copy	28.7 (0.9)	28.6 (0.8)	28.0 (0.9)	0.163	0.850	0.002	0.075
ROCF_Recall	12.2 (1.2)	14.7 (1.0)	16.5 (1.2)	3.124	0.046	0.032	0.595
ROCF_TimeCopy	2.1 (0.1)	2.2 (0.1)	2.0 (0.1)	0.087	0.917	0.001	0.063
Symbol Search	33.2 (1.7)	31.5 (1.4)	37.3 (1.6)	3.451	0.034	0.035	0.642
Decoding_FDT	17.6 (0.8)	20.1 (0.7)	18.0 (0.8)	3.124	0.046	0.032	0.595
Retrieving_FDT	20.5 (1.1)	22.8 (1.0)	20.6 (1.1)	1.539	0.217	0.016	0.324
Inhibiting_FDT	32.8 (1.7)	34.0 (1.5)	30.9 (1.7)	0.918	0.401	0.009	0.207
Shifting_FDT	43.2 (2.2)	46.5 (1.9)	40.8 (2.1)	2.023	0.135	0.021	0.414
Zoo Map test	12.4 (0.6)	12.8 (0.5)	11.6 (0.6)	0.893	0.411	0.009	0.203

d2_TR = total number of items answered; d2_TA = number of items answered correctly; d2_O = errors of omission committed; d2_C = commission errors made; d2_TOT = number of elements processed minus the total number of errors committed; d2_CON = number of relevant elements marked minus the number of commissions; d2_VAR = variation index d2; DSF = Digit Span Forward; DSB = Digit Span Backward; DSS = Digit Span Sequencing; ROCF_Copy = direct scoring in the copy phase of the Rey–Osterrieth Complex Figure; ROCF_Recall = direct scoring in the delayed Recall phase of the Rey–Osterrieth Complex Figure; Symbol Search = direct scoring of correctly answered items; Decoding_FDT = time in seconds to read all numeric items; Retrieving_FDT = time in seconds to read all non-numeric items; Inhibiting_FDT = time in seconds to read numeric items; Shifting_FDT = time in seconds to read non-numeric items; Zoo Map Test = direct score in carrying out the planning test.

**Table 4:** Effect of Val158Met genotype on neurocognitive performance and executive functions in pain-free control women.

	Val/Val (n = 20)	Val/Met (n = 35)	Met/Met (n = 9)				
	Mean (SD)	Mean (SD)	Mean (SD)	F	*p*	n^2^p	β − 1
d2_TR *	393.8 (18.2)	441.8 (13.7)	355.1 (27.1)	5.034	0.007	0.050	0.812
d2_TA *	125.8 (7.9)	158.0 (5.9)	136.7 (11.7)	5.591	0.004	0.055	0.853
d2_O	38.7 (6.6)	29.5 (4.9)	14.2 (9.8)	2.150	0.119	0.022	0.437
d2_C	4.0 (1.4)	5.0 (1.1)	0.4 (2.1)	1.800	0.168	0.018	0.373
d2_TOT *	351.0 (17.3)	407.0 (12.9)	340.7 (25.6)	4.770	0.010	0.047	0.789
d2_CON	120.4 (8.6)	151.4 (6.4)	136.4 (12.8)	4.171	0.017	0.042	0.730
d2_VAR	14.6 (1.5)	14.9 (1.1)	15.7 (2.2)	0.076	0.927	0.001	0.061
DSF	8.0 (0.4)	8.3 (0.3)	8.1 (0.6)	0.090	0.914	0.001	0.064
DSB	7.3 (0.3)	7.7 (0.2)	8.7 (0.5)	2.178	0.116	0.022	0.442
DSS	7.5 (0.4)	8.4 (0.3)	8.6 (0.6)	1.511	0.223	0.015	0.319
ROCF_Copy	30.0 (0.9)	28.1 (0.7)	31.5 (1.4)	2.693	0.070	0.027	0.529
ROCF_Recall	15.4 (1.2)	15.0 (0.9)	17.0 (1.8)	0.450	0.638	0.005	0.123
ROCF_TimeCopy	1.9 (0.1)	1.9 (0.1)	1.7 (0.2)	0.140	0.869	0.001	0.071
Symbol Search	31.2 (1.7)	33.0 (1.3)	36.4 (2.5)	1.387	0.252	0.014	0.296
Decoding_FDT	18.3 (0.8)	16.8 (0.6)	18.7 (1.2)	1.472	0.232	0.015	0.312
Retrieving_FDT	21.6 (1.1)	19.8 (0.8)	20.2 (1.7)	0.767	0.466	0.008	0.179
Inhibiting_FDT	33.0 (1.7)	32.3 (1.3)	35.1 (2.6)	0.445	0.642	0.005	0.122
Shifting_FDT	40.4 (2.2)	40.3 (1.6)	44.1 (3.3)	0.550	0.578	0.006	0.140
Zoo Map test	10.6 (0.6)	12.5 (0.5)	10.4 (1.0)	3.502	0.032	0.035	0.648

d2_TR = total number of items answered; d2_TA = number of items answered correctly; d2_O = errors of omission committed; d2_C = commission errors made; d2_TOT = number of elements processed minus the total number of errors committed; d2_CON = number of relevant elements marked minus the number of commissions; d2_VAR = variation index d2; DSF = Digit Span Forward; DSB = Digit Span Backward; DSS = Digit Span Sequencing; ROCF_Copy = direct scoring in the copy phase of the Rey–Osterrieth Complex Figure; ROCF_Recall = direct scoring in the delayed Recall phase of the Rey–Osterrieth Complex Figure; Symbol Search = direct scoring of correctly answered items; Decoding_FDT = time in seconds to read all numeric items; Retrieving_FDT = time in seconds to read all non-numeric items; Inhibiting_FDT = time in seconds to read numeric items; Shifting_FDT = time in seconds to read non-numeric items; Zoo Map Test = direct score in carrying out the planning test. * Statistically significant different by genotype (*p* < 0.015).

## Data Availability

Materials and analysis code for this study are not available in any repository; however, we will make our data accessible upon request to the corresponding author.
